# Characteristics and Risk Factors of* Helicobacter pylori* Associated Gastritis: A Prospective Cross-Sectional Study in Northeast Thailand

**DOI:** 10.1155/2016/9130602

**Published:** 2016-03-02

**Authors:** Taweesak Tongtawee, Soraya Kaewpitoon, Natthawut Kaewpitoon, Chavaboon Dechsukhum, Wilairat Leeanansaksiri, Ryan A. Loyd, Likit Matrakool, Sukij Panpimanmas

**Affiliations:** ^1^Department of Surgery, Institute of Medicine, Suranaree University of Technology, Nakhon Ratchasima 30000, Thailand; ^2^Suranaree University of Technology Hospital, Nakhon Ratchasima 30000, Thailand; ^3^Family Medicine and Community Medicine, Suranaree University of Technology, Nakhon Ratchasima 30000, Thailand; ^4^Parasite Research Unit, Suranaree University of Technology, Nakhon Ratchasima 30000, Thailand; ^5^Faculty of Public Health, Vongchavalitkul University, Nakhon Ratchasima 30000, Thailand; ^6^School of Pathology, Institute of Medicine, Suranaree University of Technology, Nakhon Ratchasima 30000, Thailand; ^7^School of Preclinic, Institute of Science, Suranaree University of Technology, Nakhon Ratchasima 30000, Thailand

## Abstract

*Background and Aim*. Risk factors for* Helicobacter pylori* infection are genetic susceptibility and poor living conditions. This study aimed to investigate the* Mdm2* gene, clarithromycin resistance, and possible risk factors for* Helicobacter pylori* infection.* Methods*. Risk factors and clinical characteristics were analyzed, including patient demographic data, patient income, personal history, possible source of transmission, patient symptoms, endoscopic findings, patterns of clarithromycin resistance, and patterns of* Mdm2* SNIP309.* Results*. Ingestion of pickled fish (OR = 11.27, 95% CI = 4.31–29.45, *p* < 0.0001), salt crab (OR = 8.83, 95% CI = 1.99–39.14, *p* < 0.001), and Papaya salad (OR = 8.73, 95% CI = 4.54–16.79, *p* < 0.01). The prevalence of clarithromycin resistance was 56% (wild type, A2143/2142A, is 23.8%; mutation, A2143/2142CG, is 35.7%; wild type + mutation is 40.5%). The genetic polymorphisms of* Mdm2* SNIP309 were SNIP309 T/T homozygous in 78%, SNIP309 G/T heterozygous in 19%, and SNIP309 G/G homozygous in 3%.* Conclusion*. Pickled fish, salt crab, and Papaya salad are positive risk factors. There was high prevalence of clarithromycin resistance. The* Mdm2* SNIP309 G/G homozygous genotype might be a risk factor for gastric cancer and the fact that it is infrequent in Thailand.

## 1. Introduction

Since the discovery of* Helicobacter pylori *in 1983, strong evidence has indicated that* H. pylori *infection plays an important role in the pathogenesis of chronic gastritis, peptic ulcer disease, and gastric malignancy [1, 2]. The risk factors for* H. pylori *infection in both developing and developed countries are closely related to poor living conditions and genetic susceptibility [3]. Low socioeconomic status, poor hygiene conditions, overcrowding, bed sharing, interfamilial clustering, family history of parental gastric disease, and person-to-person contact through fecal-oral or oral-oral contamination may be the route of transmission [4–10]. Among different Asian countries,* H. pylori *infections are more frequent in developing countries such as India, Bangladesh, Pakistan, and Thailand [11–13]. In contrast, in more industrialized and developed regions of Asia like Japan, China, and Singapore, the frequency of* H. pylori *infection has been reported to be somewhat lower [14]. The prevalence of* H. pylori *infection varies between different geographic locations, including Thailand. The prevalence of* H. pylori *infection in the south region of Thailand (14.4%) was the lowest compared with the northeast (60.6%), north (46.9%), and central (39.0%) regions (all *p* < 0.001) [15]. The northeast region of Thailand shows the highest rate of* H. pylori *infection. The primary resistance rate of* H. pylori *to clarithromycin is different in each region of the world. The overall global resistance from a systemic review in 2004 was 9.9% (95% CI: 8.3–11.7) [16]. According to a nationwide survey of* H. pylori *antibiotic resistance in Thailand, antibiotic resistance was present in 50.3% of cases including amoxicillin (5.2%), tetracycline (1.7%), clarithromycin (3.7%), metronidazole (36%), ciprofloxacin (7.7%), levofloxacin (7.2%), and multiple drugs (4.2%) with unknown mutation patterns of drug resistance [17]. Several methods have been proposed to increase the eradication rate, including the extension of the treatment duration to 14 days, the use of a four-drug regimen (bismuth-containing quadruple, sequential, and concomitant treatments), and the use of novel antibiotics, such as levofloxacin [18–21]. The progressive loss of efficacy of standard eradication therapies has made the treatment of* H. pylori* more challenging than ever. Endoscopic-guided antibiotic susceptibility testing had previously been suggested to guide treatment after failure of second-line therapies. However, its role has expanded over the years, in accordance with the current Maastricht Guidelines. Several authors have dealt with this topic, developing both efficacy trials and cost-effectiveness trials against resistant* H. pylori* infections as well as infections in naïve patients. However, results are not homogeneous enough to provide definitive advice, because antibiotic resistance is not the only reason for treatment failure. Moreover, the culture-guided approach is surrounded by many practical issues, such as the availability of both endoscopy units and microbiology laboratories and the need for a standard of quality that cannot be satisfied everywhere. Finally, pretreatment susceptibility testing should be part, and not the only weapon, of a targeted, personalized strategy to overcome* H. pylori* infection [22]. The results of our previous study showed that adding a probiotic can improve* H. pylori *eradication rates [23, 24]. Furthermore, several studies and meta-analyses have reported that certain probiotic strains can exhibit inhibitory activity against* H. pylori* bacteria. In addition, some probiotic strains can reduce the occurrence of side effects due to antibiotic therapy and consequently increase the* H. pylori* eradication rate [25–27]. In addition, geographical differences can also impair efficacy rates of different therapies, as assessed in a recent meta-analysis which showed that geographic weighting could be the main factor affecting the lack of differences between sequential and 14-day triple therapy outcomes [28].* H. pylori* infection plays an important role in gastric cancer but there is a low incident of gastric cancer in the Thai population in the setting of high prevalence of* H. pylori *infection. This unexpectedly low rate may be influenced by Thai genetic predispositions to cancer.* Mdm2 *is the major negative regulator of* p*53, the key tumor suppressor involved in the tumorigenesis of the majority of human cancers.* Mdm2 *is proposed to regulate* p*53 at the posttranslational level by enhancing* p*53 degradation through* E*3 ligase activity [29–31]. The clinical data concerning the role of* Mdm2 *SNIP309 in gastric cancer development is limited. A case-control study among the Iranian gastric cancer population showed that* Mdm2 *SNIP309 is a risk factor for this cancer with an odds ratio of 2.08 (95% confidence interval = 1.37–4.34). The same trend was observed in the Chinese [32, 33]. This study aimed to investigate the characteristics of* H. pylori *associated gastritis, clarithromycin resistance,* Mdm2 *polymorphisms, and significant risk factors of* H. pylori *associated gastritis among the Thai population.

## 2. Materials and Methods

### 2.1. Patients

Three hundred patients undergoing esophagogastroduodenoscopy for investigation of dyspeptic symptoms participated in this study from June 2014 to June 2015. The following exclusion criteria were applied: age below 18 or above 70 years old, previous* H. pylori *eradication treatment prior to the previous 2 months, significant medical illnesses, history of previous gastric surgery, and the use of antimicrobials or gastrointestinal medications like PPIs or bismuth compounds within the previous 2 months. The study was performed in accordance with good clinical practice and the guidelines of the Declaration of Helsinki. All patients provided written informed consent and the study protocol was approved by the Ethics Committee for Research Involving Human Subjects, Suranaree University of Technology (EC-57-22 and EC-57-34).

### 2.2. Diagnosis of* H. pylori *Associated Gastritis

A diagnosis of* H. pylori *associated gastritis was made if* H. pylori *were seen on histopathological examination and the rapid urease test was positive. Finally, we proved bacterial infection by PCR.

### 2.3. Biopsy Specimens

Biopsy was done according to the Updated Sydney classification [34], which indicates sampling from 5 biopsy sites. Each specimen was obtained from each of the following locations: the lesser curvature of the corpus about 4 cm proximal to the angularis (1), the lesser curvature (2) and the greater curvature of the antrum (3) both within 2 to 3 cm of the pylorus, the middle portion of the greater curvature of the corpus, approximately 8 cm from the cardia (4), and the incisura angularis (5).

### 2.4. Esophagogastroduodenoscopy (EGD)

Local anesthesia was the same as that for conventional gastroscopy. The gastroscopic procedures were performed using an upper GI video endoscope (Olympus EVIS EXERA III, CV-190). The whole stomach was examined first with conventional endoscopy and then biopsies were performed. A symptom questionnaire (abdominal pain, vomiting, diarrhea, gastrointestinal bleeding, and iron deficiency anemia) was completed by the patient at the time of initial EGD in the endoscopy room (timeout).

### 2.5. Histological Analysis

Gastric tissue specimens for histological analysis were sent to the pathologists. The hematoxylin and eosin stain and Giemsa stain were used for identification of* H. pylori*. The pathological analysis was made by 5 pathologists at Bangkok Pathological Laboratory outside Suranaree University of Technology.

### 2.6. DNA Isolation Method

The DNA of* H. pylori *was extracted from frozen gastric tissue biopsy specimens, which were stored at a temperature of less than –20°C, using the QIAamp DNA FFPE tissue kit (Qiagen, USA). DNA extraction was performed according to the manufacturer protocol. Briefly, ten tissue sections of 5 *μ*M thick were collected in 1.5 mL microcentrifuge tubes. The tissue specimens were placed in a microcentrifuge tube, and buffer ATL (180 *μ*L) and proteinase K (20 *μ*L) were added. The samples were mixed by vortexing and incubated at 56°C until the tissues were completely lysed. Buffer AL (200 *μ*L) was added to the samples, which were subsequently incubated at 70°C for 10 minutes. Next, 240 *μ*L of 100% ethanol was added to the samples, which were mixed by vortexing for 15 seconds. Each sample was placed in a QIAamp spin column and centrifuged at 8000 rpm for 1 minute. The columns were washed with AW1 buffer (500 *μ*L), and samples were centrifuged at 8000 rpm for 1 minute. AW2 buffer (500 *μ*L) was added to the column, and samples were centrifuged at 14,000 rpm for 3 minutes. Buffer AE (200 *μ*L) was added to each sample, and samples were incubated for 1 minute prior to centrifugation at 8000 rpm for 1 minute. Finally, the DNA was extracted from the tissue.

### 2.7. Real-Time PCR Hybridization Probe Methods for 23S rRNA Gene Point Mutation

The mutation detection of the 23S rRNA gene was performed by using the real-time PCR technique for template amplification. A hybridization fluorescent probe was utilized for PCR product detection. The real-time PCR procedure was accomplished by using the Light Cycler® 480 instrument (Roche Diagnostics, Neuilly sur Seine, France). The identification of target PCR products was accomplished by melting curve analyses. The target PCR products were amplified by using the primers HPYS and HPYA as reported in the previous literature. PCR-RFLP can also detect the point mutation A2142C of the 23S rRNA gene associated with resistance of* H. pylori *to clarithromycin. The amplified products have a size of 267 bp. The hybridization probes include the one that is in the mutation sites of the 23S rRNA gene of* H. pylori*, the sensor probe. The sequence is 5-GGCAAGACGGAAAGACC-3, nucleotides 2504 to 2520. This sensor probe is labeled by LC-red 640 at 5′ and phosphorylated at 3′. The anchor probe hybridizes to the PCR product at the site 3 bp upstream to the sensor probe. The probe sequence is 5-TGTAGTGGAGGTGAAAATTCCTCCTACCC-3, nucleotides 2473 to 2501, GenBank accession number U27270. The probe is labeled with fluorescein at 3′. 3 *μ*L DNA templates were subjected to PCR reaction in the final volume of 20 *μ*L. The reaction mixture consists of MgCl_2_ (25 mM), forward and reverse primers (20 M each), sensor and anchor probes (20 M each), and 2 *μ*L of FastStart DNA Master Hybridization Probes (Roche Diagnostics). PCR amplification comprised an initial denaturation cycle at 95°C for 10 min, followed by 50 amplification cycles (with a temperature transition rate of 20°C/s) consisting of 95°C for 0 s, annealing at 60°C for 10 s, and extension at 72°C for 17 s. After amplification, a melting step was performed, consisting of 95°C for 0 s, cooling to 45°C for 30 s (with a temperature transition rate of 20°C/s), and finally a slow rise in the temperature to 85°C at a rate of 0.1°C/s with continuous acquisition of fluorescence decline. According to previous reports using this real-time PCR protocol, this melting curve analysis can detect the possible three mutant genotypes along with the wild type according to different Tm. The reported Tm of the wild type, A2121C, A2142G, and A2143G were 61.5, 58.0, 53, and 53.6°C, respectively.

### 2.8. Real-Time PCR Hybridization Probe Methods for* Mdm2 *SNIP309 Genotypes

*Mdm2 *SNIP309 genotypes were analyzed using real-time PCR. The hybridization probes (light-cycler probe) were utilized for this analysis. The real-time PCR procedure was accomplished by using the Light Cycler® 480 instrument (Roche Diagnostics, Neuilly sur Seine, France). The identification of target PCR products was accomplished by melting curve analyses. The target PCR products were amplified by using the primers as reported in the previous literature. The hybridization probes included the one that is in the SNIP309 (the sensor probe). This sensor probe was labeled by LC-red 640 at 5′ and phosphorylated at 3′. The anchor probe hybridizes to the PCR product at the site 3 bp upstream to the sensor probe. 3 *μ*L DNA templates were subjected to PCR reaction in the final volume of 20 *μ*L. The reaction mixture consisted of MgCl_2_ (25 mM), forward and reverse primers (20 M each), sensor and anchor probes (20 M each), and 2 *μ*L of FastStart DNA Master Hybridization Probes (Roche Diagnostics). PCR amplification comprised an initial denaturation cycle at 95°C for 10 min, followed by 50 amplification cycles (with a temperature transition rate of 20°C/s) consisting of 95°C for 0 s, annealing at 60°C for 10 s, and extension at 72°C for 17 s. After amplification, a melting step was performed, consisting of 95°C for 0 s, cooling to 45°C for 30 s (with a temperature transition rate of 20°C/s), and finally a slow rise in the temperature to 85°C at a rate of 0.1°C/s with continuous acquisition of fluorescence decline. The genotype of each patient was categorized into the three genotypes SNIP309 G/G homozygous, T/T homozygous, and G/T heterozygous based on the different melting curves.

### 2.9. Risk Factors, Endoscopic Finding, Personal History, and Possible Route of Transmission for* H. pylori *Infection

The following variables collected in the questionnaire were analyzed as possible risk factors for* H. pylori *infection: age, gender, and patient's income. The clinical symptoms experienced during the study included abdominal pain, vomiting, diarrhea, GI bleeding, and iron deficiency anemia. Endoscopic findings included gastric ulcer, duodenal ulcer, gastric and duodenal ulcer, inflammatory polyp, nonulcer gastritis, nonulcer duodenitis, and gastroesophageal reflux disease. Possible sources of transmission included family history, ingesting food from street vendors, being a farmer, nonvegetarian food, ingesting pickled fish, ingesting salt crab, ingesting Papaya salad, or ingesting Thai vermicelli with curry. Personal history included smoking, alcohol ingestion, high temperature food intake, and spicy food.

### 2.10. Statistical Analysis

The associations of patient's demographic data, patient's symptom, endoscopic finding, possible source of transmission, and personal history with* H. pylori *positive results were examined through univariate analysis. Backward stepwise procedures were used to build the multivariate analysis; the final model included only those variables that were found to be statistically significant in the univariate analysis. The associations were expressed as odds ratios (OR) with their confidence intervals (95% CI). The data were analyzed with the SPSS software (SPSS for Windows, version 16). Significance was set at *p* < 0.05.

## 3. Results

**Figure 1:**
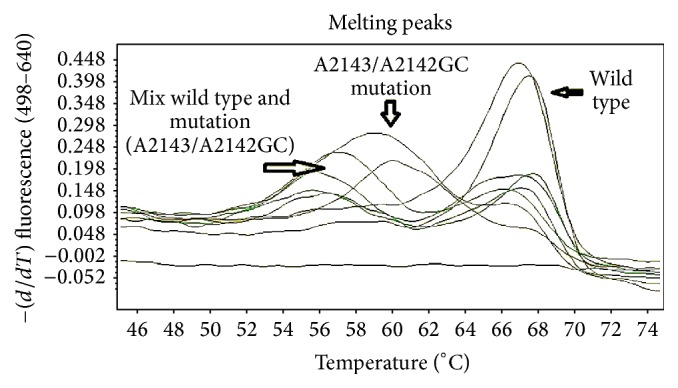
Pattern of clarithromycin resistance by using the real-time PCR hybridization probe method.

**Figure 2:**
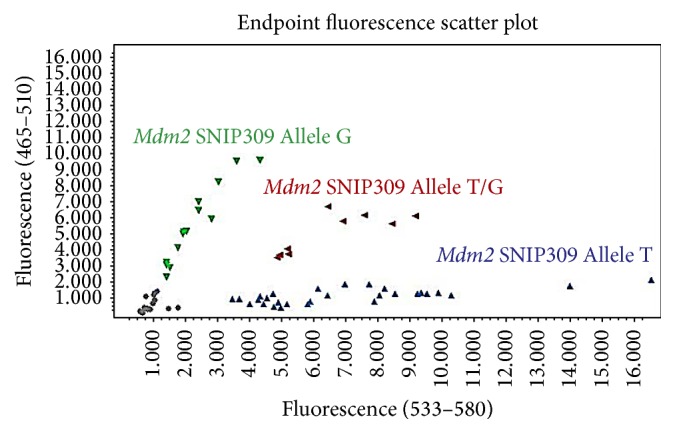
Pattern of genetic* Mdm2* SNIP309 polymorphism using real-time PCR hybridization probes (light-cycler probe).

A total of 300 patients were enrolled in this study [153 males (51%) and 149 females (49%)] from the northeast regions of Thailand. The total number of* H. pylori *infected individuals was 150 and the total number of noninfected individuals was also 150. Looking at the patients' demographic data and* H. pylori *infection by univariate analysis showed no association between them (Table 1). This study showed a high rate of 23S ribosomal RNA point mutations (56.2%). Among the mutations group, the rates of cases which had the wild type genotype, mutant strain, and mixed wild type and mutant genotype were 23.8%, 35.7%, and 40.5%, respectively (Table 2 and Figure 1). The incidence of* Mdm2 *SNIP309 T/T homozygous was 78% and that of* Mdm2 *SNIP309 G/T heterozygous was 19% and that of* Mdm2 *SNIP309 G/G homozygous was 3%. The results show that the* Mdm2 *SNIP309 T/T and* Mdm2 *SNIP309 G/T genotypes are rather high in this Thai population (Table 3 and Figure 2).

### 3.1. Patient's Symptom, Endoscopic Finding, and* H. pylori *Infection

The associations between patient's symptoms, endoscopic findings, and* H. pylori *infection were analyzed by using univariate analysis. The results showed an association only between the patient's symptoms and* H. pylori *infection (*p* < 0.01) but no association was found by using the binary logistic regression model. Abdominal pain and iron deficiency anemia had this relative association with* H. pylori *infection (Table 4).

### 3.2. Possible Source of Transmission, Personal History, and* H. pylori *Infection

The associations between the possible source of transmission, personal history, and* H. pylori *infection were analyzed by using univariate analysis. The results showed an association only between the source of transmission and* H. pylori *infection (*p* < 0.01) (Table 5). The results showed ingestion of pickled fish, salt crab, or Papaya salad to be significant predictors for* H. pylori *infection (*p* < 0.001) (Table 6). Subjects who ingested pickled fish were 11.27 times more likely to have* H. pylori *infection compared with subjects who did not. Those who ingested salt crab were 8.83 times more likely to have* H. pylori *infection compared with those who did not, and those who ingested Papaya salad were 8.73 times more likely to have* H. pylori *infection compared with those who did not (Table 7).

## 4. Discussion

The prevalence of* H. pylori *infection varies between different geographic locations, including Thailand. The highest prevalence of* H. pylori *infection is found in the northeast (60.6%) [15]. The reasons are unclear and limited studies were reported from Thailand. Some studies reported that risk factors for* H. pylori *infection are generally considered to include lower education level and low annual income [35, 36]. The fecal-oral and oral-oral routes are important transmission routes of* H. pylori *and the oral cavity is a potential extragastric reservoir for* H. pylori* [37]. Some popular foods found in the northeast region of Thailand including pickled fish, salt crab, and Papaya salad were found to be significant predictors of* H. pylori *infection in our area by both univariate and binary logistic regression model analysis. To answer this question, we plan to try to culture* H. pylori *from these suspected foods. Age group, patient's income, and personal history were not associated with* H. pylori *infection in our area. Abdominal pain and iron deficiency anemia were the most common symptoms in the patients with* H. pylori *infection whereas duodenal ulcer and nonulcer gastritis were the most common endoscopic findings in our patient population from northeast Thailand but neither was statistically associated with* H. pylori *infection in northeast Thailand. According to our results, the majority of histologically proven* H. pylori *infected cases have mutant genotype, which confers clarithromycin resistance; the clinical data indicates that most of these cases have a poor response to treatment with standard regimen. This observation indicates that, in the cases that have resistant strains, this treatment protocol (clarithromycin base triple therapy) is ineffective to eradicate the bacteria in our area and provide clinical practice outcomes. The possible reasons that underlie the mixed genotypes are multiple infections of the same patient by two strains or the occurrence of a mutation after infection by a single strain. Further genotypic analyses are necessary to confirm these possible mechanisms and larger multicenter studies about mutation patterns are needed to test this hypothesis. Physicians should be concerned about the local resistance prevalence in their area and choose the most appropriate regimen for* H. pylori *eradication.

Cancer of the stomach is the fifth most common human cancer worldwide. Intriguingly, marked variation in the gastric cancer incidence is observed. The highest incidence of gastric cancer is found in Asia such as Korea (41.8/100,000) whereas the lowest incidence is found in Africa and Northern America. In the Thai population, the incidence of gastric cancer is only 3.5/100,000. As chronic gastritis is the major predisposing condition to gastric cancer, study of the association between these conditions has become an area of interest. However, in Thailand, where the gastric cancer incidence falls into the low-risk country group, the prevalence of* H. pylori *associated gastritis is intriguingly high especially in the northeast region. Thailand is an area of the enigmatic situation that is also reported in Africa [38]. Many studies have investigated the association between* H. pylori *infection and gastric cancer in Asia. A study from India failed to demonstrate an association between* H. pylori *infection and gastric cancer [39–42] whereas studies from China and Japan demonstrated an association between* H. pylori *infection and gastric cancer [43, 44]. Our study suggests that the frequency of* Mdm2 *SNIP309 G/G is very low among the Thai population, which can explain to some extent the low incidence of gastric cancer changes.* Mdm2 *SNIP309 T/T homozygous and* Mdm2 *SNIP309 G/T heterozygous are both rather high in prevalence. According to previous data, these genotypes confer a protective effect for certain human cancers including gastric cancer and may provide the answer to the “Thailand enigma.” This study reported several lifestyle-related risk factors for stomach cancer in northeast Thailand [45].* H. pylori *infection was associated with variables indicative of ingestion of some popular foods such as pickled fish, salt crab, and Papaya salad. These are favorite foods in the northeast and north regions of Thailand including Laos PDR. Unfortunately, there is lack of data on the clarithromycin resistance, gastric cancer, and* H. pylori *infection prevalence in Laos PDR which has a similar lifestyle to the northeast region of Thailand. In conclusion,* H. pylori *infection was associated with variables indicative of ingestion of some popular foods. There was high prevalence of clarithromycin resistance. Therefore, the use of clarithromycin-based triple therapy is not recommended as an empiric first-line regimen for* H. pylori *eradication in our area. The* Mdm2 *SNIP309 G/G homozygous genotype might be a risk factor for gastric cancer and the fact that it is infrequent in Thailand could explain to some extent the low incidence of gastric cancer in the Thai population. Larger multicenter studies are needed to test this hypothesis.

## Figures and Tables

**Table 1:** Association between patient's demographic data and *H. pylori* infection (univariate analysis).

Variable	Positive for *H. pylori*, *n* (%)	Negative for *H. pylori*, *n* (%)	*p* value^*∗*^
	Total number: 150	Total number: 150	
Age group			0.91
17–30	41 (27.3)	28 (18.6)	
31–44	30 (20)	31 (20.6)	
45–58	41 (27.3)	43 (28.6)	
59–70	38 (25.3)	48 (32)	
Sex (male/female)	72/78	81/69	0.96
Income			0.97
<5,000 Baht/month	5 (3.3)	19 (12.7)	
5,000–10,000 Baht/month	86 (57.3)	69 (46)	
10,000–15,000 Baht/month	50 (33.3)	56 (37.3)	
>15,000 Baht/month	9 (6)	6 (4)	

^*∗*^Significance is set at *p* < 0.05.

**Table 2:** Mutation patterns of 23S ribosomal RNA point mutations.

Test susceptible/resistant to clarithromycin	*n* = 168
Wild type, A2143/2142A (susceptible)	23.8%

Mutation, A2143/2142CG (resistant)	35.7%

Wild type + mutation (susceptible + resistant)	40.5%

**Table 3:** *Mdm2* SNIP309 polymorphism.

*Mdm2* SNIP309 polymorphism	*n* = 300
SINP309 T/T homozygous	78%

SNIP309 G/T heterozygous	19%

*Mdm2* G/G homozygous	3%

**Table 4:** Association between patient's symptoms, endoscopic findings, and *H. pylori* infection (univariate analysis).

Variable	Positive for *H. pylori*, *n* (%)	Negative for *H. pylori*, *n* (%)	*p* value^*∗*^
	Total number: 150	Total number: 150	
Patient's symptom			<0.01^*∗*^
Abdominal pain	73 (48.7)	68 (45.3)	
Gastrointestinal bleeding	12 (8)	12 (8)	
Vomiting	8 (5.3)	11 (7.3)	
Diarrhea	3 (2)	10 (6.7)	
Iron deficiency anemia	54 (36)	49 (32.7)	
Endoscopic finding			0.17
Gastric ulcer	7 (4.7)	12 (8)	
Duodenal ulcer	22 (14.7)	13 (8.7)	
Gastric and duodenal ulcer	3 (2)	12 (8)	
Inflammatory polyp	8 (5.3)	9 (6)	
Nonulcer gastritis	95 (63.3)	84 (56)	
Nonulcer duodenitis	1 (0.7)	11 (7.3)	
Gastroesophageal reflux disease	14 (9.3)	9 (6)	

^*∗*^Significance is set at *p* < 0.05.

**Table 5:** Predictive value of patient's symptoms for *H. pylori* infection (multivariate analysis).

Variable	95% confidence		
	Odds ratio	Interval	*p* value
Abdominal pain	1.11	0.57–1.41	0.64
Gastrointestinal bleeding	0.91	0.40–2.08	0.83
Vomiting	0.64	0.25–1.63	0.35
Diarrhea	0.28	0.07–1.06	0.06
Iron deficiency anemia	1.15	0.72–1.86	0.54

Significance is set at *p* < 0.05.

**Table 6:** Association between possible source of transmission, personal history, and *H. pylori* infection (univariate analysis).

Variable	Positive for *H. pylori*, *n* (%)	Negative for *H. pylori*, *n* (%)	*p* value^*∗*^
	Total number: 150	Total number: 150	
Possible root of transmission			<0.01^*∗*^
Family history	2 (1.3)	14 (9.3)	
Ingesting food from street vendor	15 (10)	23 (15.3)	
Being a farmer	4 (2.7)	35 (23.3)	
Nonvegetarian food	—	28 (18.7)	
Ingesting pickled fish	42 (28)	5 (5.3)	
Ingesting salt crab	16 (10.7)	2 (1.3)	
Ingesting Papaya salad	68 (45.3)	13 (8.7)	
Thai vermicelli eaten with curry	3 (2)	30 (20)	
Personal history			0.05
Smoking	49 (32.7)	20 (13.3)	
Alcohol drinking	89 (59.3)	30 (20)	
High temperature food intake	5 (3.3)	65 (43.3)	
Spicy food	7 (4.7)	35 (23.3)	

^*∗*^Significance is set at *p* < 0.05.

**Table 7:** Predictive value of possible source of transmission for *H. pylori* infection.

Variable	95% confidence		
	Odds ratio	Interval	*p* value^*∗*^
Family history	0.13	0.02–0.58	0.08
Ingesting food from street vendor	0.58	0.29–1.16	0.12
Being a farmer	0.09	0.03–0.26	<0.01^*∗*^
Ingesting pickled fish	11.27	4.31–29.45	<0.01^*∗*^
Ingesting salt crab	8.83	1.99–39.14	<0.01^*∗*^
Ingesting Papaya salad	8.73	4.54–16.79	<0.01^*∗*^
Thai vermicelli eaten with curry	0.08	0.02–0.27	<0.01^*∗*^

^*∗*^Significance is set at *p* < 0.05.
